# Ecosystem effects of fishing & El Niño at the Galápagos Marine Reserve

**DOI:** 10.7717/peerj.6878

**Published:** 2019-05-08

**Authors:** Tyler D. Eddy, Alan M. Friedlander, Pelayo Salinas de León

**Affiliations:** 1 Changing Ocean Research Unit, Institute for the Oceans & Fisheries, University of British Columbia, Vancouver, BC, Canada; 2 Department of Biology, Dalhousie University, Halifax, NS, Canada; 3 Department of Marine Sciences, Charles Darwin Research Station, Puerto Ayora, Galápagos Islands, Ecuador; 4 Nereus Program, Baruch Institute for Marine & Coastal Sciences, University of South Carolina, Columbia, SC, USA; 5 Pristine Seas, National Geographic Society, Washington, DC, USA; 6 Fisheries Ecology Research Lab, University of Hawai’i, Honolulu, HI, USA

**Keywords:** Ecopath with Ecosim, Ecosystem-based fisheries management, *Myctereoperca olfax*, Fishers’ ecological knowledge, Keystone species, Grouper

## Abstract

The Galápagos Archipelago is home to a diverse range of marine bioregions due to the confluence of several cold and warm water currents, resulting in some of the most productive tropical marine ecosystems in the world. These ecosystems are strongly influenced by El Niño events which can reduce primary production by an order of magnitude, dramatically reducing energy available throughout the food web. Fisheries are an important component of the local economy, although artisanal and illegal overfishing have dramatically reduced the productivity of invertebrate and finfish resources in recent decades, resulting in reductions in catches for local fishers. The regionally-endemic sailfin grouper (*Myctereoperca olfax*), locally known as *bacalao*, was once the most important fished species in the Galápagos, but is now listed as vulnerable by the IUCN due to its limited range and dramatic declines in catch over time. It is unknown how reduction of this predatory species has affected ecosystem structure and function. In the absence of stock assessments, we used an estimate of unfished *bacalao* biomass from fishers’ ecological knowledge along with unfished biomass estimates of other heavily exploited stocks—lobster (*Panulirus penicillatus* and *P. gracilis*) and sea cucumber (*Isostichopus fuscus*)—to create historical, unfished versions of existing modern day ecosystem models. We used the unfished and modern versions of the ecosystem models to test the ecosystem effects of *bacalao* exploitation at the Bolivar Channel, located in the cold, west upwelling bioregion of the archipelago during both El Niño and non El Niño years, and at Floreana Island, in the warmer, central bioregion. Fishers’ ecological knowledge indicates that at present, the biomass of *bacalao* is at least seven times lower than when unfished. This reduced *bacalao* biomass is linked with a greatly reduced ecosystem role compared to when unfished, and ecosystem role is further reduced in El Niño years. Allowing *bacalao* populations to rebuild to at least half of unfished biomass would partially restore their role within these ecosystems, while also resulting in greater fisheries catches. Comparing ecosystem impacts caused by fishing and El Niño, fishing has had a greater negative impact on *bacalao* ecosystem role than regular El Niño events.

## Introduction

Understanding how environmental factors interact with exploitation of resources is a common concern among natural resource managers, biological conservationists, and scientists (Bender, Case & Gilpin, 1984). In the marine environment, it has been suggested that fishing magnifies fluctuations in natural populations due to removing older individuals of the population which changes demographic parameters such as intrinsic growth rates (Anderson et al., 2008). Removing top predators and keystone species from ecosystems can also cause trophic cascades and reshuffling of ecosystems (Worm et al., 2009; Smith et al., 2011; Collie et al., 2016). We are now in the Anthropocene and changes in environmental conditions are the greatest that they have ever been (Bopp et al., 2013), therefore, it is important to understand how populations are affected by the interaction between resource use and a changing world (Cheung, Reygondeau & Frölicher, 2016).

The Galapágos Archipelago hosts ecologically important, diverse, and fragile ecosystems with high levels of endemism (Edgar et al., 2010). The strong upweling of the eastward-flowing Cromwell Current on the western portion of the archipelago produces some of the world’s most productive tropical waters (Wolff, Ruiz & Taylor, 2012). This region is strongly affected by El Niño events when upweling slows causing water temperatures to increase and substantially reducing primary production flow through ecosystems resulting in dramatic declines in abundance of many species (Edgar et al., 2010; Wolff, Ruiz & Taylor, 2012).

For local people living in the Galapágos, fisheries are an important source of income. Landed fish are sold within the Archipelago to supply the rapidly growing tourism industry, as well as exported. The handline fishery for the regionally-endemic sailfin grouper (*Mycteroperca olfax*; locally referred to as *bacalao*) has operated since the 1920s and was once the most valuable fishery in the Galápagos, comprising over 40% of the mixed whitefish catch (Reck, 1983). More recently, *bacalao* has accounted for <10% of the finfish catch, with fishers indicating that catch rates and average individual size have declined significantly over time (Bustamante, 1998; Burbano et al., 2014; Schiller et al., 2015). Recent data indicate that 96% of *bacalao* caught are below reproductive size, with few large spawning individuals, and an extremely skewed sex ratio, suggesting eminent reproductive failure (Usseglio et al., 2016). Historical photographs from 1925 to 1938 indicate that average size of caught *bacalao* has declined from 77 to 47 cm in 2012 (Usseglio et al., 2016). The economic value of the fishery for *bacalao* has decreased tremendously over time and is not as economically important today as the fisheries for lobster (locally referred to as *langosta*; *Panulirus penicillatus* and *P. gracilis*), sea cucumber (locally referred to as *pepino*; *Isostichopus fuscus*) or pelagic fishes—mainly wahoo, yellowfin tuna, and swordfish (*Acanthocybium solandri*, *Thunnus albacares*, and *Xiphias gladius*, respectively).

In 1998, the Special Law of Galápagos established the Galápagos Marine Reserve, which excluded industrial fishing fleets within 40 nautical miles of the islands. The coastal zoning scheme for the Galápagos Marine Reserve implemented in 2000, closed some areas to fishing (approximately 20% of the coastline, however <1% of the entire Galápagos Marine Reserve), however compliance among fishers is poor—with many fishers claiming to not know the locations of protected areas—and there is little to no enforcement of closed areas (Castrejón & Charles, 2013; Usseglio, Schuhbauer & Friedlander, 2014). Socio-economic inputs dominated the selection process of no-take zones in the Galápagos Marine Reserve, with ecological considerations having a lower priority (Edgar et al., 2004; González et al., 2008; Castrejón & Charles, 2013). Other than no-take zones, there are no specific regulations for managing *bacalao* or any other finfish species within the Galápagos Marine Reserve.

The IUCN Red List currently categorizes *bacalao* as vulnerable due to its limited range and declining fishery catches (Erisman & Craig, 2018). The distribution of *bacalao* is limited to the offshore islands of Cocos, Costa Rica; Malpelo, Colombia; and Galápagos, Ecuador in the Tropical Eastern Pacific, however, it is rare at both Cocos and Malpelo (Marconi, Beck & Majluf, 2001). *Bacalao* is a top predator in the coastal ecosystems of the Galápagos and is a sequential hermaphrodite, as all individuals are born as females, and transition to males at approximately 12 years of age and 80 cm total length (Usseglio et al., 2015). The life history characteristics that make *bacalao* susceptible to overfishing, its ecological role within the Galápagos ecosystem, and its current threatened conservation status have prompted recent efforts to inform sustainable management of *bacalao* (Salinas-de-León, Rastoin & Acuña-Marrero, 2015; Usseglio et al., 2015, 2016).

Understanding the impacts of fisheries and environment on marine ecosystems is a central tenant of ecosystem-based fisheries management (Pikitch et al., 2004; Browman et al., 2004; Link, 2010). Ecosystem models are one tool that have been used extensively to understand ecosystem effects of fishing (Christensen & Walters, 2004; Worm et al., 2009; Smith et al., 2011; Collie et al., 2016), as well as to understand climate change impacts on marine ecosystems (Brown et al., 2010; Cornwall & Eddy, 2015; Payne et al., 2016, Cheung, Reygondeau & Frölicher, 2016). It is also important to understand how fisheries and environmental effects on ecosystems have changed through time, as exploited populations can have reduced roles within ecosystems—especially for keystone groups (Coll, Palomera & Tudela, 2009; Eddy et al., 2014). In the absence of detailed historical fisheries data, historical ecology, catch reconstructions, and fishers’ ecological knowledge are tools that can be used to estimate the biomass of unfished populations (Jackson et al., 2001; Johannes, Freeman & Hamilton, 2000; Rosenberg et al., 2005; Lotze et al., 2006; Pauly & Zeller, 2016).

Here, we use estimates of unfished *bacalao*, *langosta*, and *pepino* biomass to understand the magnitude of depletion throughout history. We employ existing ecosystem models for the Bolivar Channel (Ruiz & Wolff, 2011; Wolff, Ruiz & Taylor, 2012), in the cold western bioregion, and Floreana Island, in the warmer central bioregion (Okey et al., 2004), to run simulations of varying levels of *bacalao* fishing for unfished and modern periods. We then examine the impacts of *bacalao* fishing on its biomass and ecosystem role, as well as its impacts on other groups in the ecosystem and how these impacts change during El Niño years at the Bolivar Channel.

## Methods

### Ecosystem models

**Figure 1:**
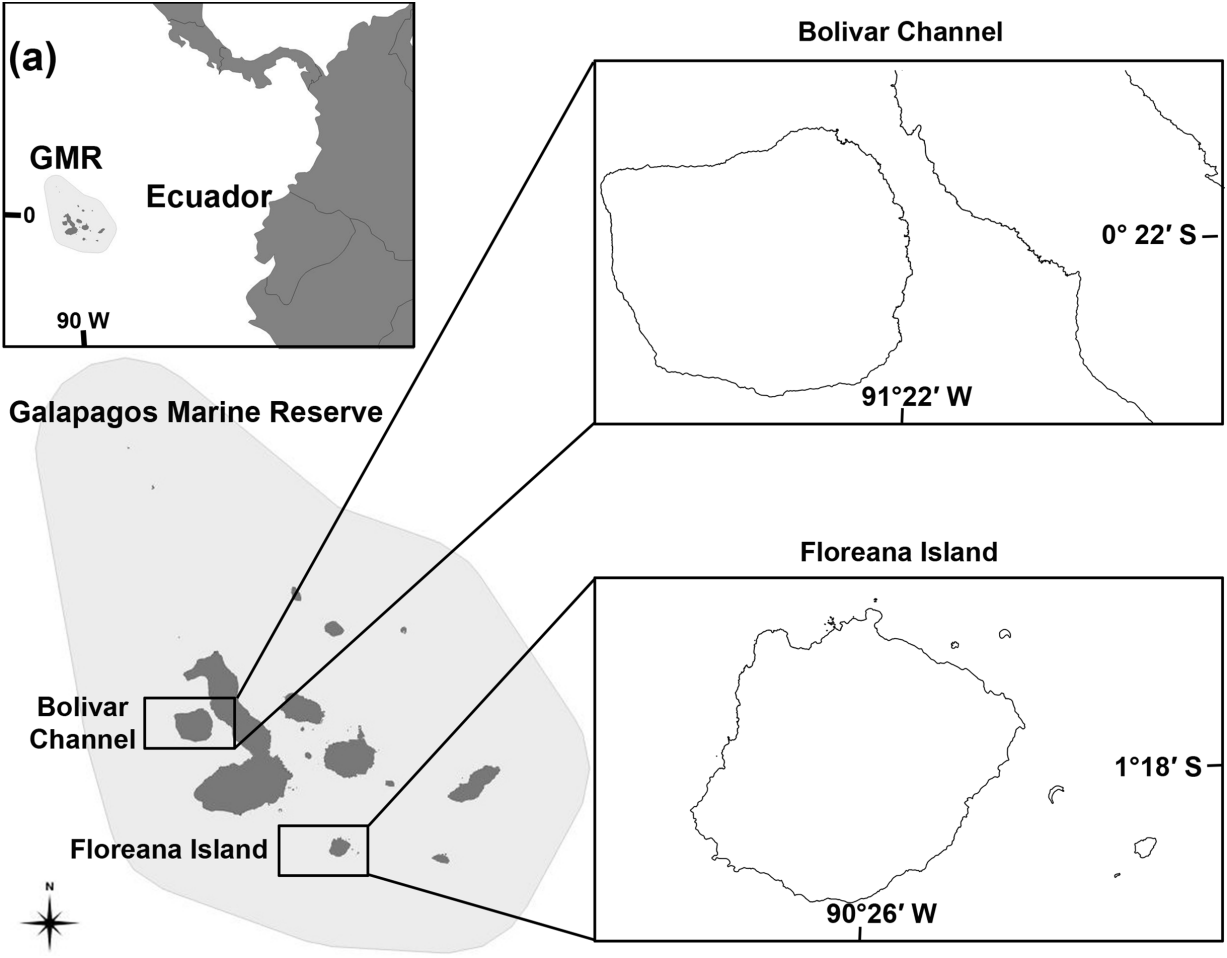
Map of the Galápagos Marine Reserve (GMR) indicating ecosystem model locations at the Bolivar Channel and Floreana Island.

We used published Ecopath with Ecosim (EwE; Christensen & Walters, 2004; Christensen et al., 2008) models for Floreana Island (Okey et al., 2004) located in the warm, central bioregion and two models for Bolivar Channel, located in the cold, west bioregion, representing normal, and El Niño years (Ruiz & Wolff, 2011; Wolff, Ruiz & Taylor, 2012; Fig. 1). All three of these models include fisheries for *bacalao*. The Floreana Island model is available online from Ecobase (http://sirs.agrocampus-ouest.fr/EcoBase/), while the Bolivar Channel models are available by contacting the model developers.

#### Floreana Island

Floreana Island is located in the warm, central bioregion, characterized by a high regional mix of cold and warm-water biota (Fig. 1; Okey et al., 2004). The Floreana Island ecosystem model was parameterized for the rocky reefs surrounding the island between 0 and 20 m depth (partial depth range inhabited by *bacalao*), covering 6.44 km^2^ or 36% of the rocky reef habitat of the island. The model was parameterized for the time period 2000–2001, and is characterized by 42 functional groups including *bacalao—*estimated to have a trophic level of 4.2 (Table 1)—with prey groups that consisted of planktivorous reef fishes accounting for 60% of diet, small benthic invertebrate eating fishes accounting for 20% of diet, and 20% of diet represented by imports and informed by the literature (Okey et al., 2004).

**Table 1:** Floreana Island ecosystem model characteristics and fishing simulation results.

	Functional group	TL	Prey groups	Unfished	Modern
1	Sharks	4.4	3–12, 14, 17–19, 21, 22, 27, 29, 32–36	+	–
2	Toothed cetaceans	4.4	1, 3, 5–11, 21, 27, 32	‐	‐
3	Bacalao	4.2	12, 14	‐‐‐	‐‐‐
4	Birds	4.1	3, 6, 7, 9–12, 14, 15, 18, 21, 27, 32	‐	‐
5	Sea lions	4.0	3, 6, 8–10, 12, 14, 21, 27	+	+
6	Pelagic predators	3.9	7, 11, 18, 21, 32	+	+
7	Non-commercial reef predators	3.8	12, 14, 15, 18, 19, 21, 37, 39, 43	++	+
8	Octopods	3.5	7, 10–12, 14, 16–19, 21, 38	‐	+
9	Pelagic planktivores	3.4	15, 19, 37, 39, 40	‐	‐
10	Other commercial reef predators	3.3	11, 12, 15, 18, 19, 22, 24, 37, 39	‐	‐
11	Large benthic invertebrate eaters	3.3	13, 16–19, 21, 24, 28, 31, 35, 36, 38	‐	‐
12	Planktivorous reef fishes	3.3	15, 39, 40	+	+
13	Hexaplex gastropod	3.0	38, 43	‐	‐
14	Small benthic invertebrate eaters	3.0	15, 19, 22, 24, 25, 28, 31, 37–39, 41–43	+	+
15	Carnivorous zooplankton	2.8	39, 40	‐	‐
16	Spiny lobsters	2.8	19, 20, 22–24, 26, 28, 35, 36, 38, 41–43	‐	‐
17	Slipper lobster	2.7	12, 14, 18, 19, 21, 22, 24, 28, 35, 36, 38, 42, 43	‐	‐
18	Omnivorous reef fishes	2.7	15, 19, 23–25, 28, 37–39, 41–43	‐	‐
19	Shrimps and small crabs	2.6	12, 14, 15, 21, 23–25, 28, 37–43	‐	‐
20	Asteroids	2.5	19, 23–25, 28, 30, 38, 41–43	‐	‐
21	Other herbivorous fishes	2.4	15, 28, 37, 39, 42, 43	‐	‐
22	Eucidaris urchin	2.2	25, 42, 43	+	+
23	Anemones	2.2	13, 15, 19, 20, 22, 24–26, 28, 30–31, 34–43	+	+
24	Worms and ophioroids	2.2	15, 25, 28, 37–43	‐	‐
25	Stony corals	2.2	15, 39–43	‐	‐
26	Chitons	2.2	15, 41–43	+	‐
27	Detritivorous fishes	2.1	39–43	‐	‐
28	Small gastropods	2.1	24, 25, 37, 38, 41–43	+	+
29	Sea turtles	2.1	15, 42	+	+
30	Sea cucumbers	2.1	23, 24, 41, 43	+	+
31	Other urchins	2.0	25, 41–43	‐	‐
32	Parrotfishes	2.0	25, 41–43	+	+
33	Marine iguana	2.0	42	+	+
34	Other sea cucumbers	2.0	40, 42, 43	‐	‐
35	Tripneustes urchin	2.0	42, 43	+	+
36	Lytechinus urchin	2.0	42, 43	+	+
37	Small crustaceans	2.0	40–43	‐	‐
38	Filter + suspension feeders	2.0	40, 43	‐	‐
39	Herbivorous zooplankton	2.0	40, 43	‐	‐
40	Phytoplankton	1.0		‐	‐
41	Microphytobenthos	1.0		+	+
42	Benthic algae	1.0		+	+
43	Detritus	1.0		‐	‐

**Note:**

Floreana Island ecosystem model characteristics and results indicating direction and magnitude of change in biomass of functional groups at modern levels of *bacalao* depletion (85%) compared to unfished *bacalao* biomass (1 − (*B_i_*/*B_0_*)) for unfished and modern versions of the model (TL = trophic level; ‐ = ‐ <20% change, ‐‐ = ‐ >20% and ‐ <40% change, ‐‐‐ = >40% change; + = <20% change, ++ = >20% and <40% change, +++ = >40% change).

Floreana Island production, consumption, and diet data were mostly derived from the literature; biomass estimates of benthic, demersal, and pelagic fish groups, large invertebrates, birds, turtles, sharks, and marine mammals were based on site-specific observations; and primary production was estimated from ocean color satellite data (Okey et al., 2004).

#### Bolivar channel

The Bolivar Channel is located in the cold west bioregion, characterized by cool, nutrient rich, upwelled waters, and associated biota including dense macroalgae beds, the world’s only tropical penguin, and the flightless cormorant (Fig. 1; Ruiz & Wolff, 2011). The Bolivar Channel ecosystem model was parameterized for rocky habitat between 0 and 30 m depth surrounding Isabela and Fernandina islands, covering 44 km^2^ or 14% of the total Bolivar Channel area (Ruiz & Wolff, 2011; Fig. 1). This model represents the average ecosystem state for the years 2004–2008, and includes 30 different functional groups, including *bacalao*, with a predicted trophic level of 3.39 (Table 2), and a diet of 26% sea stars and sea urchins, 20% benthic omnivorous fishes, 9% small planktivorous reef fishes, 8% surgeonfish, chubs, and giant damselfishes, 7% mullets, 6% benthic predatory fish, 5% gorgonians, and six other groups each accounting for <5% of total diet (Ruiz & Wolff, 2011). There was also a Bolivar Channel model that was forced using a 16-year time series of satellite-derived phytoplankton biomass from 1994 to 2009, which included the strong El Niño of 1997–1998 (Wolff, Ruiz & Taylor, 2012; Table 2). The El Niño version of the model has a primary productivity reduction of 76% relative to the non El Niño model (Wolff, Ruiz & Taylor, 2012).

**Table 2:** Bolivar Channel ecosystem model characteristics and fishing simulation results.

	Functional group	TL	Prey groups	Direction and magnitude of biomass change			
				Unfished non El Niño	Modern non El Niño	Unfished El Niño	Modern El Niño
1	Phytoplankton	1.00		+	-	-	-
2	Macroalgae + others	1.00		+	+	-	-
3	Surgeonfishes, chubs and giant damiselfishes	2.00	2, 30	+	+	++	+
4	Sea cucumbers and other	2.00	30	+	+	+	+
5	Herbivorous zooplankton	2.00	1, 2, 30	-	-	-	-
6	Sea turtles and marine iguanas	2.01	2, 5, 30	-	-	-	-
7	Small herbivorous gastropods	2.01	2, 5, 30	- -	-	-	-
8	Sponges and polychaetes	2.01	1, 5, 18, 30	-	+	-	-
9	Gorgonians	2.07	1, 5, 18, 30	+	-	++	+
10	Parrotfishes	2.11	2, 9, 30	- -	+	-	-
11	Mullets	2.11	1, 5, 30	++	+	+++	+
12	Benthic omnivorous fishes	2.14	1, 2, 5, 30	++	+	++	+
13	Anemones and zooanthids	2.14	1, 5, 18, 30	-	+	- -	-
14	Sea stars and sea urchins	2.28	2, 8, 9, 13, 19, 30	-	-	+	-
15	Planktivorous reef fishes	2.34	1, 5, 18, 30	+	+	+++	+
16	Small planktivorous reef fishes	2.47	1, 5, 18, 30	-	-	++	+
17	Lobsters	2.64	2, 14, 30	-	-	+	+
18	Predatory zooplankton	2.80	1, 5, 30	-	-	- -	-
19	Large gastropods and other sea stars	2.92	2, 7, 9, 13, 14, 20, 30	+	+	+	+
20	Small predatory gastropods	2.96	5, 7–9, 13, 18, 30	+	+	++	+
21	Small benthic predatory fishes	3.19	5, 11, 12, 14, 16, 18	+++	++	+++	++
22	Benthic predatory fishes	3.36	3, 7, 11, 12, 14, 16, 17, 20, 21, 23, 24	+++	+	++	+
23	Barracudas	3.37	3, 11, 12, 15, 16, 21, 22	+++	+	+++	+
24	Groupers	3.39	3, 7, 11, 12, 14, 15–17, 20–23	- - -	- - -	- - -	- - -
25	Jacks and mackerels	3.40	3, 11, 16	- -	-	+++	+
26	Rays	3.43	4, 14, 17, 19	-	-	-	-
27	Predatory marine mammals	3.49	10, 11, 12, 15–17, 19, 21–25, 29	+++	+	+++	-
28	Seabirds	3.50	3, 11, 16, 23	-	-	+++	+
29	Sharks	3.89	6, 11, 12, 16, 21–23, 25–27	+++	+	+++	+
30	Detritus	1.00		-	+	-	-

**Note:**

Bolivar Channel ecosystem model characteristics and results indicating direction and magnitude of change in biomass of functional groups at modern *bacalao* level of depletion (85%) compared to unfished *bacalao* biomass (1 − (*B_i_*/*B_0_*)) for unfished and modern versions of the El Niño and non El Nino models (TL = trophic level; (TL = trophic level; - = - <20% change, - - = - >20% and - <40% change, - - - = >40% change; + = <20% change, ++ = >20% and <40% change, +++ = >40% change).

Bolivar Channel production, consumption, and diet parameters were estimated from the literature, while biomass estimates of fish, invertebrate, sea bird, marine mammal, and reptile were based on observations from within the model area (Ruiz & Wolff, 2011; Wolff, Ruiz & Taylor, 2012). Primary production estimates were derived from satellite observations of ocean color, while zooplankton biomass was estimated from observational data (Ruiz & Wolff, 2011; Wolff, Ruiz & Taylor, 2012).

### Unfished biomass estimates

To understand the ecosystem impact of *bacalao* fishing through time in the absence of stock assessments, we estimated unfished biomass of the most heavily exploited species (*bacalao, langosta*, and *pepino*), using a variety of sources. Burbano et al. (2014) interviewed fishers of different ages, asking them to recall their best catches of *bacalao*. Older fishers indicated a 6.82-fold decline in their best catches from the 1940–1950s compared to 2010. Ruttenberg (2001) surveyed reef fishes using underwater visual surveys in the Galápagos Marine Reserve at both lightly and heavily fished areas in 1998, and found 22-fold less biomass in heavily fished areas. Schiller et al. (2015) performed catch reconstructions at the Galápagos Islands, however did not provide specific values of catches or biomass of *bacalao*. *Pepino* were estimated at six- to eightfold greater biomass when unfished (Okey et al., 2004) and there has been a 10-fold decrease in catch per unit effort (CPUE; Defeo, Castilla & Castrejón, 2009). *Langosta* are reported to be 10-fold greater in the more remote northern Darwin and Wolf Islands (Edgar et al., 2010). We applied the *bacalao* multiplier of 6.82 from Burbano et al. (2014) to the modern biomass values to estimate unfished biomasses in each of the three ecosystem models. We applied unfished biomass multipliers of seven for *pepino* (mean value from Okey et al., 2004) and 10 for *langosta* (Edgar et al., 2010).

### Modeling strategy

We simulated a range of *bacalao* fisheries mortality rates (*F*)—from no exploitation (*F* = 0) to local extinction—using the Floreana Island and Bolivar Channel models to understand the ecosystem effects of fishing (*sensu* Eddy et al., 2015). For the modern models, we held fishing mortality (*F*) of all other exploited groups constant at their most recent levels. For the unfished models, we removed all fishing morality aside from that for *bacalao.* Simulations were run until the end of their historical time series when available, and then run for 100 years to allow models to reach equilibrium following the change in *bacalao* fishing mortality. Additionally, in order to understand the ecosystem effects of *bacalao* exploitation during El Niño years, we ran a similar range of exploitation rates of *bacalao* using the Bolivar Channel El Niño model. Only the Bolivar Channel El Niño model was fit to time series data to tune vulnerability values. In the absence of model fits to time series for the other two models, we used the default vulnerabilities.

In order to standardize exploitation levels across all models, we calculated the level of *bacalao* depletion as the proportion of biomass compared to unfished biomass $\left( {1 - \left( {{B_i}/{B_0}} \right)} \right)$. We report ecosystem effects for 60% depletion as this is a common target used in fisheries management (Worm et al., 2009; Smith et al., 2011). We calculated maximum sustainable yield (MSY) as the maximum *bacalao* catch produced by any simulation.

### Ecosystem impacts

We calculated the ecosystem impacts of varying levels of *bacalao* exploitation on the biomasses of all other functional groups in the ecosystem. We represent the biomasses of the other functional groups as the proportion of their biomass when *bacalao* was fished at the level of depletion *i* (*B_i_*) compared to the biomass when *bacalao* was unfished (*B*_0_). This impact is analgous to the interaction strength index developed by Okey (2004) presented in the original Floreana Island EwE model description (Okey et al., 2004).

Keystone species are those that display disproportionately large impacts on the biomasses of other groups in an ecosystem compared to their biomass (Power et al., 1996). Keystone values of *bacalao* in each of three models for each time period were calculated by EwE using the mixed trophic analysis which quantifies how much other functional groups are impacted in their biomass (Christensen & Walters, 2004) and the keystone index developed by Libralato, Christensen & Pauly (2006) which quantifies how the ecosystem impacts relate to the biomass of the impacting group. The Okey (2004) dynamic keystoneness index more explicitly accounts for the relative biomass of the impacting group, whereas the Libralato, Christensen & Pauly (2006) keystone index tends to emphasize interaction strength. We compare the results from both approaches in the discussion.

### Sensitivity analysis

To assess how sensitive model results were to parameter estimates, we performed a sensitivity analysis on *bacalao* unfished biomass estimates and *bacalao* diet. For the three unfished models, instead of using the 6.82 *bacalao* biomass multiplier, we used a conservative estimate of half of the biomass multiplier of 3.41. Using this conservative estimate, we ran the same fishing simulations as above. We compared the results of these simulations to the results from the original unfished models and calculated sensitivity as the difference in ecosystem impacts that resulted from 100% *bacalao* depletion (local extinction), as these simulations produced the greatest ecosystem impacts—measured as biomass changes in other functional groups. As indicated above, using the multiplier of 22 times more *bacalao* biomass in unfished versions of the model did not produce simulations that reached equilibrium. We also examined the effect of switching *bacalao* diets from the Floreana model to the Bolivar Channel models and vice versa. The only stomach content analysis of *bacalao* indicates that it is piscivorous (Rodriguez, 1984). The Floreana *bacalao* diet is piscivorous, therefore the Bolivar Channel model was run with the Floreana diet to represent a piscivorous *bacalao* diet. We examined model sensitivity in the same way as for biomass, and also by comparing the estimated trophic levels of *bacalao* with each diet. The Bolivar Channel El Niño model parameterized with the Floreana diet did not reach equilibrium during simulations and therefore results are not presented.

## Results

### Ecosystem role of *bacalao*

**Figure 2:**
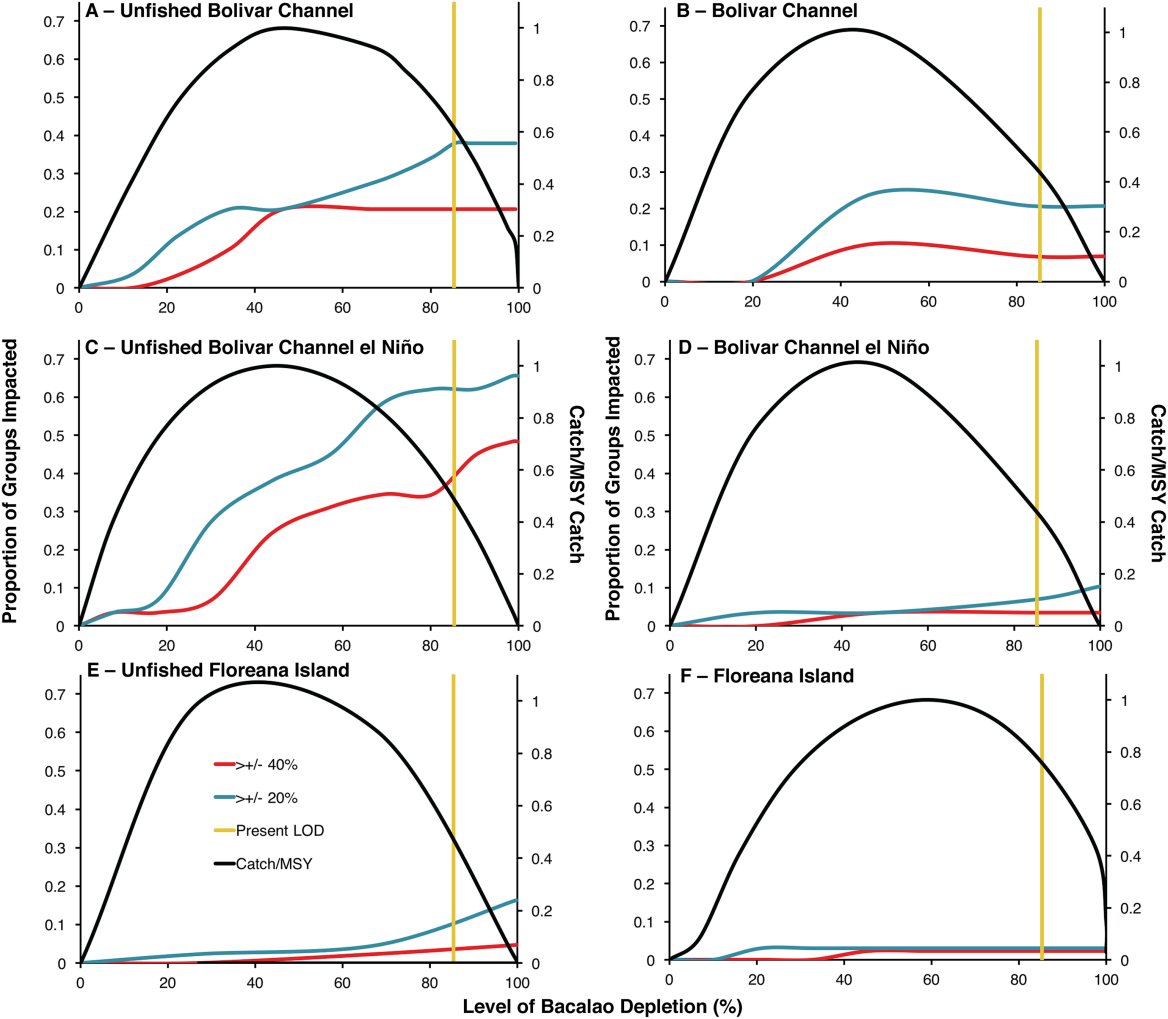
Trade-offs between ecosystem impacts and catches of *bacalao* as a function of fishing exploitation for different EwE models during historical and modern times. Ecosystem impact is represented as the proportion of trophic groups other than *bacalao* with a biomass change of by at least 20% (blue line) or 40% (red line) of their unfished biomass. Modern level of *bacalao* depletion (LOD) is represented by the yellow line. Catch is plotted on the secondary axis as a proportion of maximum sustainable yield (MSY). (A) Unfished Bolivar Channel; (B) Bolivar Channel; (C) Unfished Bolivar Channel El Niño; (D) Bolivar Channel El Niño; (E) Unfished Floreana Island; (F) Floreana Island.

Unfished biomass versions of the Bolivar Channel El Niño and non El Niño ecosystem models indicated much greater ecosystem impacts of *bacalao* exploitation compared to the modern day models (Fig. 2). In the unfished non El Niño model, at the modern level of *bacalao* depletion (85%), 38% of functional groups were impacted by at least a 20% change in biomass, while 21% of functional groups were impacted by at least a 40% change in biomass (Table 1; Fig. 2A). In the modern day model, at the current level of *bacalao* depletion (85%), 21% and 7% of functional groups were impacted by at least 20% and 40% biomass, respectively (Table 1; Fig. 2B). There was an even greater impact of *bacalao* exploitation in the unfished El Niño version of the Bolivar Channel model, where at 85% *bacalao* depletion, 62% and 34% of functional groups were impacted by at least 20% and 40% of their biomass, respectively, compared to when *bacalao* was unfished (Table 1; Figs. 2C and 2D). The modern day version of the model also showed much smaller ecosystem impacts, similar to the modern day non El Niño model at modern level of depletion (85%), with 7% and 3% of functional groups impacted by 20% and 40% of biomass compared to when *bacalao* was unexploited (Table 1; Figs. 2B and 2D).

The functional groups that differed in biomass the most at modern *bacalao* level of depletion (85%) in the unfished non El Niño Bolivar Channel model compared to the modern day version with increased biomasses were: phytoplankton, gorgonians, mullets, benthic omnivorous fishes, small benthic predatory fishes, benthic predatory fishes, predatory marine mammals, and sharks (Table 1). Groups that showed decreased biomasses in their unfished non El Niño model compared to the modern day version were: small herbivorous gastropods, sponges and polychaetes, parrotfishes, anemones and zooanthids, jacks and mackerels, and detritus (Table 1). Comparing the two El Niño Bolivar Channel models at modern *bacalao* levels of depletion, functional groups that were at greater biomass in the unfished model compared to the modern day version were: surgeonfishes, chubs and giant damiselfishes, gorgonians, mullets, benthic omnivorous fishes, sea stars and sea urchins, planktivorous reef fishes, small planktivorous reef fishes, small predatory gastropods, small benthic predatory fishes, barracudas, jacks and mackerels, predatory marine mammals, seabirds, and sharks (Table 1). Functional groups that showed the greatest biomass decreases were: anemones and zooanthids and predatory zooplankton (Table 1).

The unfished *bacalao* biomass version of the Floreana Island model showed greater influence of *bacalao* compared to the modern version (Table 2; Figs. 2E and 2F). In the unfished biomass model, at 85% *bacalao* depletion, 5% of functional groups were impacted by at least 20% biomass, while 2% were impacted by 40% of their biomass (Table 1; Fig. 2E). Only 3% and 2% of groups were impacted by both 20% and 40% of their biomass, respectively in the modern day model when *bacalao* was depleted by 85% (Table 2; Fig. 2F). The functional groups most impacted by 85% *bacalao* depletion in the unfished biomass model were sharks, non-commercial reef predators, and chitons which were all at greater biomasses compared to modern day, while octopods had lower biomass in the unfished model (Table 2).

### Tradeoffs between *bacalao* catch and ecosystem impacts

The modern level of *bacalao* exploitation of 85% depletion is higher than that predicted to yield MSY in all six ecosystem models (Fig. 2). Unfished biomass MSY was predicted to occur at lower levels of exploitation in the El Niño Bolivar Channel and Floreana models (44% and 38% depletion, respectively) compared to the modern day versions (45% and 58% depletion, respectively; Figs. 2C–2F). The non El Niño Bolivar Channel models predicted MSY to occur at 45% depletion at unfished biomass and 43% in the modern day (Figs. 2A and 2B). In all of the unfished models, fishing at levels producing MSY compared to the modern day exploitation rate (vertical yellow lines) reduces ecosystem impacts, and increases fisheries catches (Fig. 2). The reduction of ecosystem impacts by reducing fishing depletion to MSY levels is greatest for the unfished Bolivar Channel models (Figs. 2A and 2C). In the unfished Floreana model, reducing fishing depletion to MSY levels results in decrease in functional groups impacted by both 40% and 20% of their biomass (Fig. 2F). In the modern versions of all models, the ecosystem role of *bacalao* has been diminished due to greater levels of depletion (Figs. 2B, 2D and 2F). In the Bolivar Channel models, reducing fishing pressure to MSY levels decreases ecosystem impacts (Figs. 2A–2D).

It is clear that the reduction of *bacalao* biomass due to fishing represented in the modern models has resulted in a diminished ecosystem role (Fig. 2). By comparing the Bolivar Chanel models during El Niño and non El Niño years and during unfished and modern states, it is possible to weigh the relative effects of fishing through top-down trophic control and environmental conditions that affect bottom-up primary production (Fig. 2). Comparatively, the El Niño models do show opposite responses in the unfished and modern models, whereby ecosystem effects of *bacalao* exploitation get amplified during El Niño unfished, while in the modern Bolivar Channel models, ecosystem impacts of *bacalao* exploitation are diminished during El Niño (Figs. 2A–2D).

### Keystone role of *bacalao*

**Figure 3:**
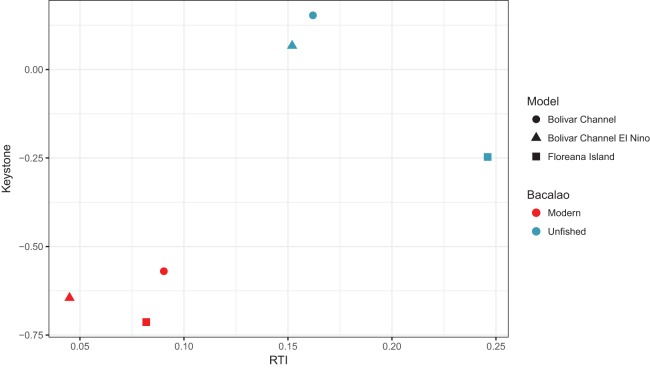
Keystone values (Libralato, Christensen & Pauly, 2006) for *bacalao* during unfished and modern time periods for the Galápagos EwE models. RTI, relative total impact.

In the unfished versions of all three models, the keystone role (Libralato, Christensen & Pauly, 2006) of *bacalao* was greater than in modern versions of the models (Fig. 3). Of the unfished models, *bacalao* showed the greatest keystone value in the Bolivar Channel, followed by the El Niño version of the Bolivar Channel, and Floreana Island (Fig. 3). For the modern day models, this ranking remained the same, however, all of the values were lower, indicating a diminished keystone role (Fig. 3).

### Sensitivity analysis

Reducing unfished *bacalao* biomass reduced the ecosystem effects of *bacalao* exploitation (Table 3). The greatest reduction in ecosystem impacts was observed for the Bolivar Channel El Niño model, which also showed the greatest ecosystem impacts during simulations with the greater estimate of unfished *bacalao* biomass (Fig. 2). Similarly, for the unfished Bolivar Channel and Floreana models, reduced *bacalao* biomass also reduced ecosystem impacts (Table 3; Fig. 2). Swapping the Floreana and Bolivar Channel *bacalao* diets led to increased ecosystem impacts in both cases. Swapping diets reduced *bacalao* trophic level at Floreana decreased from 4.2 to 4.0, while it increased *bacalao* trophic level at Bolivar Channel from 3.4 to 3.5.

**Table 3:** Sensitivity analysis results from reducing unfished *bacalao* biomass and from switching *bacalao* diets from the Floreana model to the Bolivar Channel models and vice versa.

Sensitivity scenario	Per cent change in number of functional groups impacted by >±40% of their biomass	Per cent change in number of functional groups impacted by >±20% of their biomass
Bolivar Channel unfished with reduced *bacalao* biomass	−10%	−24%
Bolivar Channel El Niño unfished with reduced *bacalao* biomass	−24%	−24%
Floreana unfished with reduced *bacalao* biomass	−2%	−12%
Bolivar Channel with Floreana diet	+7%	+7%
Floreana with Bolivar diet	0	+4%

**Note:**

Results are from simulations that produced the maximum ecosystem impacts (100% *bacalao* depletion).

## Discussion

### Impact of *bacalao* fishing

We have shown how the ecosystem role of *bacalao* has been greatly diminished with overexploitation—which has depleted the stock by 85% compared to unfished levels. This reduced biomass has led to decreases in the ecosystem role played by *bacalao* in all models examined, through direct and indirect feeding interactions with other groups. We did not observe any local extinctions of other species when *bacalao* was fished to local extinction, however, there were large changes in biomass of many functional groups, indicating large ecosystem reshuffling. Our results also suggest that *bacalao* catches could be much greater if their populations were allowed to recover to approximately half of their unfished biomass. This recovery would also reduce ecosystem impacts, creating a win-win situation. The cost associated with such a management action would be a short-term reduction in fisheries catches, which would allow stocks to recover to levels that produce MSY. Such trade-offs among ecological, economical, and societal factors are a central tenant of fisheries policy and management, and have been examined in detail in many marine ecosystems (Worm et al., 2009; Smith et al., 2011; Eddy et al., 2017).

### Ecosystem role of groupers

Our results indicate that when unfished, *bacalao* has a much greater keystone role in all three models, also reflected by greater ecosystem effects when unfished with dynamic simulations of *bacalao* exploitation. A low *bacalao* keystone value was also reported for the modern Floreana Island model using a different keystone index, adding confidence to our findings (Okey, 2004; Okey et al., 2004). Recent work indicates that *bacalao* are extremely size over-fished, suggesting that their modern diet could be different than historically (Usseglio et al., 2016). These observations are consistent with other studies that have quantified the change in keystone role of species resulting from intensive exploitation, such as dolphins in the northwest Mediterranean (Coll, Palomera & Tudela, 2009), and lobster (*Jasus edwardsii*) in New Zealand (Eddy et al., 2014). For lobster in New Zealand, the increase in keystone role resulted from a density-dependent change in their diet in unfished areas where greater lobster biomass was observed, where they were more generalist and herbivorous, resulting in decreased trophic level (Freeman, 2007; Jack, Wing & McLeod, 2009). Currently, it is unclear how the diet of *bacalao* at the Galápagos may have changed since historical times when they were found in much higher abundance, but previous work has shown *bacalao* to be highly piscivorous (Rodriguez, 1984). Our sensitivity analysis indicates that a change in *bacalao* diet is a source of variation in ecosystem impacts, however not as great as impacts due to reduced biomass due to fishing.

The aim of using non-traditional, quantitative sources such as fishers’ ecological knowledge is to extend time series to avoid the “shifting baseline syndrome” (Pauly, 1995) in order to provide appropriate targets for fisheries management and conservation (Rosenberg et al., 2005; Eddy, Gardner & Pérez-Matus, 2010; Burbano et al., 2014; Eddy, Cheung & Bruno, 2018). It has been found that a greater sample size of interviewed fishers leads to less biased estimates of past fishing activities (Shepperson et al., 2014). The study that informed our unfished *bacalao* biomass estimate interviewed 124 fishers, accounting for 24% of the fishing population (Burbano et al., 2014).

Groupers are particularly vulnerable to overexploitation due to their life history characteristics (Sadovy de Mitcheson et al., 2013; Usseglio et al., 2015). A massive decline in the IUCN designated “endangered” goliath grouper (*Epinephelus itajara*) was documented in south Florida USA, using old photographs of winning catches from fishing competitions that indicated a 86% decline in grouper populations from 1956 to 1979, and newspaper accounts dating back to 1923 suggested that the population was already impacted by fishing prior to 1950 (McClenachan, 2009). The prey of goliath grouper likely benefited directly by this large reduction in predation by grouper, and we would also expect indirect negative effects on prey of grouper prey through meso-predator release, as well as increased competition among grouper prey with other species (McClenachan, 2009). In our simulations, the groups that benefitted most from *bacalao* exploitation in the Bolivar Channel were anenomes and zooanthids for both El Niño and non El Niño models; parrotfishes and sponges and polychaetes for the non El Niño model, and predatory zooplankton in the El Niño model. Following the strong 1997–1998 El Niño, anemone barrens were observed, providing new benthic habitat for colonization (Okey, Shepherd & Martínez, 2003). For Floreana Island, octopods benefitted from *bacalao* exploitation. All of these increases were due to indirect feeding interactions, highlighting ecosystem cascades that result from removing a key species.

### Unfished biomass estimates

Working with data poor study systems and trying to reconstruct historical ecosystem states often requires using qualitative information sources that have a high amount of uncertainty in parameter estimates (McClenachan, 2009; Schiller et al., 2015). We only presented results for the lower estimate of unfished *bacalao* biomass, because the higher estimate of 22-fold more *bacalao* from surveys (Ruttenberg, 2001) caused the model to become highly unbalanced, without an equilibrium being reaching during simulations. Therefore, our simulations should be considered a conservative estimate of unfished *bacalao* biomass. We acknowledge that this is one of many hypotheses of how the unfished ecosystem may have looked. Species other than those that were primarily targeted by the artisanal fishery have also been exploited, through bycatch, bait or subsistence fisheries.

### Bacalao diet estimates

The results from the food-web modeling approach that we have employed here are dependent on diet preferences specified in the models. As above, *bacalao* diet composition varies substantially between the two models. Therefore, we can think of these two different ecosystem models as alternate hypotheses of ecosystem structure and function at the Galápagos Islands, which can be further tested and refined as future data become available. It should also be noted that these two model areas are located in different bioregions with different community compositions, which likely contribute to differences in diet. Surveys do not indicate higher abundances of *bacalao* in no-take areas, likely due to lack of compliance and enforcement (Edgar et al., 2004), which has also been observed for lobster (Buglass et al., 2018). In the future, if these areas do provide *bacalao* populations protection from fishing, they will be an invaluable resource to understand not only how *bacalao* diet composition changes as biomass increases, but also how the biomasses of all other species in the ecosystem change as a result of direct and indirect interactions.

### Fisheries management in a changing world

Understanding the impact that regular, although not always predictable, environmental events, such as El Niño have on ecosystems is extremely important for ecosystem-based fisheries management, as exploitation of marine resources does not occur in isolation—these events impact species of conservation interest and species of importance for the tourism sector (Lynham et al., 2015; Salinas-de-León et al., 2016). The cold-west upwelling region of the Galápagos is particularly sensitive to El Niño events, which result in increased water temperatures and large declines in primary production (Wolff, Ruiz & Taylor, 2012) with catastrophic effects on many marine species (Okey, Shepherd & Martínez, 2003; Okey, 2004; Edgar et al., 2010). Climate change projections for the Galápagos Islands from an ensemble of Earth system models indicate an increase in primary production under the strong mitigation of emissions scenario (RCP 2.6; Bopp et al., 2013). However, the business as usual emissions scenario (RCP 8.5) indicates a decrease in primary production (Bopp et al., 2013), which could lead to an ecosystem state observed in Bolivar Channel El Niño model. Considering the relative contributions of *bacalao* fishing and environmental factors to changes in simulated ecosystem effects, our results indicate that reduced *bacalao* biomass through overexploitation has produced greater ecosystem effects than El Niño. This finding is similar to another study which concluded that the ecosystem effects of fishing greatly outweighed projected impacts of ocean acidification (Cornwall & Eddy, 2015).

*Bacalao* is not the only species that has been heavily exploited at the Galápagos, as there have been collapses of *pepino* (sea cucumber) and *langosta* (lobster) fisheries in recent history (Bustamante, Okey & Banks, 2008). Additionally, the Galápagos Archipelago is home to a number of species of conservation interest, and understanding the trophic relationships that mediate direct and indirect interactions among these species is imperative to understand how the decline or recovery of one species will affect others. This approach is the basis of ecosystem-based fisheries management, which sets the management unit at the scale of ecosystem rather than individual populations. This study, along with other recent studies describing life history (Usseglio et al., 2015), reproductive behavior (Salinas-de-León, Rastoin & Acuña-Marrero, 2015), fishery bycatch and discards rates (Zimmerhackel et al., 2015), and fisheries characteristics (Usseglio et al., 2016) of *bacalao* will allow for an evidence-based management plan for this species, to be developed in partnership with the fisheries managing authority, the Galápagos National Park Directorate, and stakeholders such as fishers, the tourism industry, and environmental NGOs.

## Conclusions

Using multiple lines of evidence about how exploited populations have changed through time, we have created hypotheses about how unfished ecosystems at the Galápagos may have been structured. The large reduction in *bacalao* biomass due to fishing has reduced its ecosystem role through direct and indirect feeding interactions. Allowing *bacalao* populations to recover to approximately half of their unfished biomass will partially restore their ecosystem roles as well as provide greater fisheries productivity.

## Supplemental Information

10.7717/peerj.6878/supp-1 Supplemental Information 1 Output simulation data. Output simulation data used for Fig. 2

10.7717/peerj.6878/supp-2 Supplemental Information 2 Keystone simulation data. Keystone simulation data used to create Fig. 3.

## Additional Information and Declarations

### Data Availability

The following information was supplied regarding data availability: We do not have permission to share the third-party models that we used to perform the simulations, however, interested parties can access the Floreana Island model on the Ecobase website – http://sirs.agrocampus-ouest.fr/EcoBase/. The Bolivar Channel models can be obtained by contacting Diego Ruiz – diego.ruiz@zmt-breman.de.

## References

[ref-1] Anderson CNK, Hsieh C-H, Sandin SA, Hewitt R, Hollowed A, Beddington J, May RM, Sugihara G (2008). Why fishing magnifies fluctuations in fish abundance. Nature.

[ref-2] Bender EA, Case TJ, Gilpin ME (1984). Perturbation experiments in community ecology: theory and practice. Ecology.

[ref-3] Bopp L, Resplandy L, Orr JC, Doney SC, Dunne JP, Gehlen M, Halloran P, Heinze C, Ilyina T, Séférian R, Tjiputra J, Vichi M (2013). Multiple stressors of ocean ecosystems in the 21st century: projections with CMIP5 models. Biogeosciences.

[ref-4] Browman HI, Cury PM, Hilborn R, Jennings S, Lotze HK, Mace PM, Murawski S, Pauly D, Sissenwine M, Stergiou KI, Zeller D (2004). Perspectives on ecosystem-based approaches to the management of marine resources. Marine Ecology Progress Series.

[ref-5] Brown CJ, Fulton EA, Hobday AJ, Matear RJ, Possingham HP, Bulman C, Christensen V, Forrest RE, Gehrke PC, Gribble NA, Griffiths SP, Lozano-Montes H, Martin JM, Metcalf S, Okey TA, Watson R, Richardson AJ (2010). Effects of climate-driven primary production change on marine food webs: implications for fisheries and conservation. Global Change Biology.

[ref-6] Buglass S, Reyes H, Ramirez-González J, Eddy TD, Salinas-de-León P, Marrin JM (2018). Evaluating the effectiveness of coastal no-take zones of the Galapagos Marine Reserve for the red spiny lobster, *Panulirus penicillatus*. Marine Policy.

[ref-7] Burbano DV, Mena CF, Guarderas P, Vinueza L, Reck R, Denkinger J, Vinueza L (2014). Shifting baselines in the Galápagos white fin fishery, using fisher’s anecdotes to reassess fisheries management: the case of the Galápagos grouper. The Galápagos Marine Reserve, Social and Ecological Interactions in the Galápagos Islands.

[ref-8] Bustamante RH, World Wildlife Fund–Fundación Natura (1998). The artisanal fishing sector of the Galápagos and the 1997 fishing season. Galápagos Report 1997–1998.

[ref-9] Bustamante RH, Okey TA, Banks S, McClanahan TR, Branch GM (2008). Biodiversity and food-web structure of a Galapagos shallow rocky-reef ecosystem. Food Webs and the Dynamics of Marine Reefs.

[ref-10] Castrejón M, Charles A (2013). Improving fisheries co-management through ecosystem-based spatial management: the Galápagos Marine Reserve. Marine Policy.

[ref-12] Cheung WWL, Reygondeau G, Frölicher TL (2016). Large benefits to marine fisheries of meeting the 1.5°C global warming target. Science.

[ref-13] Christensen V, Walters CJ (2004). Ecopath with Ecosim: methods, capabilities and limitations. Ecological Modelling.

[ref-14] Christensen V, Walters CJ, Pauly D, Forrest R (2008). Ecopath with Ecosim user guide v6. http://ecopath.org.

[ref-15] Coll M, Palomera I, Tudela S (2009). Decadal changes in a NW Mediterranean Sea food web in relation to fishing exploitation. Ecological Modelling.

[ref-16] Collie JS, Botsford LW, Hastings A, Kaplan IC, Largier JL, Livingston PA, Plagányi E, Rose KA, Wells BK, Werner FE (2016). Ecosystem models for fisheries management: finding the sweet spot. Fish and Fisheries.

[ref-17] Cornwall CE, Eddy TD (2015). Effects of near-future ocean acidification, fishing, and marine protection on a temperate coastal ecosystem. Conservation Biology.

[ref-18] Defeo O, Castilla JC, Castrejón M, Lodeiros C, Alió J, Freites L, González N, Guerra A, Rey-Méndez M (2009). Pesquerías artesanales de invertebrados en América Latina: paradigmas emergentes de manejo y gobernanza. Foro Iberoamericano de los Recursos Marinos y la Acuicultura II.

[ref-19] Eddy TD, Cheung WWL, Bruno JF (2018). Historical baselines of coral cover on tropical reefs as estimated by expert opinion. PeerJ.

[ref-20] Eddy TD, Coll M, Fulton EA, Lotze HK (2015). Trade-offs between invertebrate fisheries catches and ecosystem impacts in coastal New Zealand. ICES Journal of Marine Science.

[ref-21] Eddy TD, Gardner JPA, Pérez-Matus A (2010). Applying fishers’ ecological knowledge to construct past and future lobster stocks in the Juan Fernández Archipelago, Chile. PLOS ONE.

[ref-22] Eddy TD, Lotze HK, Fulton EA, Coll M, Ainsworth CH, De Araújo JN, Bulman CM, Bundy A, Christensen V, Field JC, Gribble NA, Hasan M, Mackinson S, Townsend H (2017). Ecosystem effects of invertebrate fisheries. Fish and Fisheries.

[ref-23] Eddy TD, Pitcher TJ, MacDiarmid AB, Byfield TT, Tam JC, Jones TT, Bell JJ, Gardner JPA (2014). Lobsters as keystone: only in unfished ecosystems?. Ecological Modelling.

[ref-24] Edgar GJ, Banks SA, Brandt M, Bustamante RH, Chiriboga A, Earle SA, Garske LE, Glynn PW, Grove JS, Henderson S, Hickman CP, Miller KA, Rivera F, Wellington GM (2010). El Niño, grazers and fisheries interact to greatly elevate extinction risk for Galápagos marine species. Global Change Biology.

[ref-25] Edgar GJ, Bustamante RH, Fariña J-M, Calvopiña M, Martínez C, Tora-Granda MV (2004). Bias in evaluating the effects of marine protected areas: the importance of baseline data for the Galápagos Marine Reserve. Environmental Conservation.

[ref-64] Erisman B, Craig MT (2018). Mycteroperca olfax. The IUCN Red List of Threatened Species.

[ref-26] Freeman DJ (2007). The ecology of spiny lobsters (*Jasus edwardsii*) on fished and unfished reefs.

[ref-27] González JA, Montes C, Rodríguez J, Tapia W (2008). Rethinking the Galápagos islands as a complex social-ecological system: implications for conservation and management. Ecology and Society.

[ref-30] Jack L, Wing SR, McLeod RJ (2009). Prey base shifts in red rock lobster *Jasus edwardsii* in response to habitat conversion in Fiordland marine reserves: implications for effective spatial management. Marine Ecology Progress Series.

[ref-31] Jackson JBC, Kirby MX, Berger WH, Bjorndal KA, Botsford LW, Bourque BJ, Bradbury RH, Cooke R, Erlandson J, Estes JA, Hughes TP, Kidwell S, Lange CB, Lenihan HS, Pandolfi JM, Peterson CH, Steneck RS, Tegner MJ, Warner RR (2001). Historical overfishing and the recent collapse of coastal ecosystems. Science.

[ref-32] Johannes RE, Freeman MMR, Hamilton RJ (2000). Ignore fishers’ knowledge and miss the boat. Fish and Fisheries.

[ref-33] Libralato S, Christensen V, Pauly D (2006). A method for identifying keystone species in food web models. Ecological Modelling.

[ref-34] Link J (2010). Ecosystem-based fisheries management: confronting tradeoffs.

[ref-35] Lotze HK, Lenihan HS, Bourque BJ, Bradbury RH, Cooke RG (2006). Depletion, degradation, and recovery potential of estuaries and coastal seas. Science.

[ref-36] Lynham J, Costello C, Gaines S, Sala E (2015). Economic valuation of marine- and shark-based tourism in the Galápagos islands: report to the Galápagos National Park. https://media.nationalgeographic.org/assets/file/GalapagosEconReport_Nov15.pdf.

[ref-37] Marconi MR, Beck S, Majluf P (2001). Conservación De Ecosistemas Transfronterizos Y Especies Amenazadas. https://www.rds.org.co/aa/img_upload/cd3189bd6b9a1ea1575134c54f92a42c/Conservaci_n_de_ecosistemas_transfronterizos_y_especies_ame.pdf.

[ref-38] McClenachan L (2009). Historical declines of goliath grouper populations in South Florida, USA. Marine Ecology Progress Series.

[ref-39] Okey TA, Okey TA (2004). A search for keystones in Prince William Sound, Alaska using a mass-continuity trophic model. Shifted Community States in Four Marine Ecosystems: Some Potential Mechanisms.

[ref-40] Okey TA, Banks S, Born AF, Bustamante RH, Calvopiña M, Edgar GJ, Eduardo Espinoza E, Fariña JM, Garske LE, Reck GK, Salazar S, Shepherd S, Toral-Granda V, Wallem P (2004). A trophic model of a Galápagos subtidal rocky reef for evaluating fisheries and conservation strategies. Ecological Modelling.

[ref-41] Okey TA, Shepherd SA, Martínez PC (2003). A new record of anemone barrens in the Galápagos. Noticias de Galapagos.

[ref-42] Pauly D (1995). Anecdotes and the shifting baseline syndrome of fisheries. Trends in Ecology & Evolution.

[ref-43] Pauly D, Zeller D (2016). Catch reconstructions reveal that global marine fisheries catches are higher than reported and declining. Nature Communications.

[ref-44] Payne MR, Barange M, Cheung WWL, MacKenzie BR, Batchelder HP, Cormon X, Eddy TD, Fernandes JA, Hollowed AB, Jones MC, Link JS, Neubauer P, Ortiz I, Queirós AM, Paula JR (2016). Uncertainties in projecting climate-change impacts in marine ecosystems. ICES Journal of Marine Science: Journal du Conseil.

[ref-45] Pikitch EK, Santora C, Babcock EA, Baku A, Bonfil R, Conover DO, Dayton P, Doukakis P, Fluharty D, Heneman B, Houde ED, Link J, Livingston PA, Mangel M, McAllister MK, Pope J, Sainsbury KJ (2004). Ecosystem-based fishery management. Science.

[ref-46] Power ME, Tilman D, Estes JA, Menge BA, Bond WJ, Mills LS, Daily G, Castilla JC, Lubchenco J, Paine RT (1996). Challenges in the quest for keystones. BioScience.

[ref-47] Reck G (1983). The coastal fisheries in the Galápagos Islands, Equador.

[ref-48] Rodriguez WT (1984). Estudio preliminar para evaluar las caracteristicas biologicas pesquerias de la *Mycteroperca ofax* en las Islas Galápagos (Equador). Instituto Nacional de Pesca Boletin Cientifico y Tecnico.

[ref-49] Rosenberg A, Bolster WJ, Alexander KE, Leavenworth WB, Cooper AB, McKenzie MG (2005). The history of ocean resources: modeling cod biomass using historical records. Frontiers in Ecology and the Environment.

[ref-50] Ruiz DJ, Wolff M (2011). The bolivar channel ecosystem of the Galápagos marine reserve: energy flow structure and role of keystone groups. Journal of Sea Research.

[ref-51] Ruttenberg BI (2001). Effects of Artisanal fishing on marine communities in the Galápagos Islands. Conservation Biology.

[ref-52] Sadovy de Mitcheson Y, Craig MT, Bertoncini AA, Carpenter KE, Cheung WWL, Choat JH, Cornish AS, Fennessy ST, Ferreira BP, Heemstra PC, Liu M, Myers RF, Pollard DA, Rhodes KL, Rocha LA, Russell BC, Samoilys MA, Sanciangco J (2013). Fishing groupers towards extinction: a global assessment of threats and extinction risks in a billion dollar fishery. Fish and Fisheries.

[ref-53] Salinas-de-León P, Acuña-Marrero D, Rastoin E, Friedlander AM, Donovan MK, Sala E (2016). Largest global shark biomass found in the northern Galápagos Islands of Darwin and Wolf. PeerJ.

[ref-54] Salinas-de-León P, Rastoin E, Acuña-Marrero D (2015). First record of a spawning aggregation for the tropical eastern Pacific endemic grouper *Mycteroperca olfax* in the Galápagos Marine Reserve. Journal of Fish Biology.

[ref-55] Schiller L, Alava JJ, Grove J, Reck G, Pauly D (2015). The demise of Darwin’s fishes: evidence of fishing down and illegal shark finning in the Galápagos Islands. Aquatic Conservation: Marine and Freshwater Ecosystems.

[ref-56] Shepperson J, Murray LG, Cook S, Whiteley H, Kaiser MJ (2014). Methodological considerations when using local knowledge to infer spatial patterns of resource exploitation in an Irish Sea fishery. Biological Conservation.

[ref-57] Smith ADM, Brown CJ, Bulman CM, Fulton EA, Johnson P (2011). Impacts of fishing low-trophic level species on marine ecosystems. Science.

[ref-58] Usseglio P, Friedlander AM, DeMartini EE, Schuhbauer A, Schemmel E, Salinas-de-León P (2015). Improved estimates of age, growth and reproduction for the regionally endemic Galápagos sailfin grouper *Mycteroperca olfax* (Jenys, 1840). PeerJ.

[ref-59] Usseglio P, Friedlander AM, Koike H, Zimmerhackel J, Schuhbauer A, Salinas de León P, Eddy TD (2016). So long and thanks for all the fish: overexploitation of the regionally endemic Galapagos grouper *Mycteroperca olfax* (Jenyns, 1840). PLOS ONE.

[ref-60] Usseglio P, Schuhbauer A, Friedlander AM, Vinueza L, Denkinger J (2014). Collaborative approach to fisheries management as a way to increase the effectiveness of future regulations in the Galápagos archipelago. The Galápagos Marine Reserve: A Dynamic Socio-Ecological System.

[ref-61] Wolff M, Ruiz DJ, Taylor M (2012). El Niño induced changes to the Bolivar Channel ecosystem (Galápagos): comparing model simulations with historical biomass time series. Marine Ecology Progress Series.

[ref-62] Worm B, Hilborn R, Baum JK, Branch TA, Collie JS, Costello C, Fogarty MJ, Fulton EA, Hutchings JA, Jennings S, Jensen OP, Lotze HK, Mace PM, McClanahan TR, Minto C, Palumbi SR, Parma AM, Ricard D, Rosenberg AA, Watson R, Zeller D (2009). Rebuilding global fisheries. Science.

[ref-63] Zimmerhackel JS, Schuhbauer AC, Usseglio P, Heel LC, Salinas-de-León P (2015). Catch, bycatch and discards of the Galápagos Marine reserve small-scale handline fishery. PeerJ.

